# Calreticulin regulates vascular endothelial growth factor-A mRNA stability in gastric cancer cells

**DOI:** 10.1371/journal.pone.0225107

**Published:** 2019-11-14

**Authors:** Po-Chu Lee, Jui-Chung Chiang, Chih-Yu Chen, Yin-Chieh Chien, Wei-Min Chen, Chin-Wei Huang, Wen-Chin Weng, Chia-I Chen, Po-Huang Lee, Chiung-Nien Chen, Hsinyu Lee

**Affiliations:** 1 Department of Traumatology, National Taiwan University Hospital, Taipei, Taiwan; 2 Department of Surgery, National Taiwan University Hospital, Taipei, Taiwan; 3 Graduate Institutes of Clinical Medicine, National Taiwan University, Taipei, Taiwan; 4 Department of Life Science, National Taiwan University, Taipei, Taiwan; 5 Department of Pediatrics, National Taiwan University Hospital, Taipei, Taiwan; 6 Department of Pediatrics, National Taiwan University, Taipei, Taiwan; 7 Department of Pediatric Neurology, National Taiwan University Children’s Hospital, Taipei, Taiwan; 8 Department of Surgery, E-DA Hospital, Kaohsiung, Taiwan; 9 Department of Electrical Engineering, National Taiwan University, Taipei, Taiwan; 10 Angiogenesis Research Center, National Taiwan University, Taipei, Taiwan; 11 Research Center for Developmental Biology, National Taiwan University, Taipei, Taiwan; 12 Regenerative Medicine and Center for Biotechnology, National Taiwan University, Taipei, Taiwan; Medical Faculty Mannheim, University of Heidelberg, GERMANY

## Abstract

Calreticulin (CRT) and vascular endothelial growth factor-A (VEGF-A) are crucial for angiogenesis, and mediate multiple malignant behaviors in gastric cancer. In this study, we report that CRT is positively correlated with VEGF-A in gastric cancer patients. Moreover, high expressions of both CRT and VEGF-A are markedly associated with the pathological stage, progression, and poor prognosis in the patients. Therefore, we sought to elucidate the mechanism by which CRT affects VEGF-A in gastric cancer. Firstly, we demonstrate the novel finding that knockdown of CRT reduced VEGF-A mRNA stability in two gastric cancer cell lines, AGS and MKN45. The AU-Rich element (ARE) is believed to play a crucial role in the maintenance of VEGF-A mRNA stability. Luciferase reporter assay shows that knockdown of CRT significantly decreased the activity of renilla luciferase with VEGF-A ARE sequence. Additionally, competition results from RNA-binding/electrophoretic mobility shift assay indicate that CRT forms an RNA-protein complex with the VEGF-A mRNA by binding to the ARE. In addition, the proliferation rate of human umbilical vein endothelial cells (HUVEC) was significantly reduced when treated with conditioned medium from CRT knockdown cells; this was rescued by exogenous VEGF-A recombinant protein. Our results demonstrate that CRT is involved in VEGF-A ARE binding protein complexes to stabilize VEGF-A mRNA, thereby promoting the angiogenesis, and progression of gastric cancer.

## Introduction

Gastric cancer is the third leading cause of cancer-related death worldwide [1], and the seventh leading cause of cancer-related mortality in Taiwan. We previously demonstrate that calreticulin (CRT) can be a prognostic marker of gastric cancer. Overexpression of CRT enhances angiogenesis and malignant behavior in gastric cancer cells, and further associated with microvessel density, tumor invasion, lymph node metastasis, and survival in patients [2]. CRT functions as a protein chaperone and regulator of Ca^2+^ homeostasis, controlling the quality of protein synthesis from endoplasmic reticulum and regulates both intracellular Ca^2+^ and cell behavior [3]. Correlations between CRT and metastasis have been reported in multiple cancers. The expression of CRT is especially higher in aggressive breast cancer cells and positively correlated with distant metastasis in tissue samples [4]. Of note, CRT has been shown previously to promote angiogenesis via activating the nitric oxide signaling pathway [5]. In particular, a positive correlation between CRT and Vascular endothelial growth factor-A (VEGF-A) has also been addressed in neuroblastoma, bladder cancer, and gastric cancer cell lines [2, 6, 7].

VEGF-A belongs to the VEGF family and is a specific ligand of the vascular endothelial growth factor receptor (VEGFR) family [8]. It is a growth and survival factor for endothelial cells, and therefore highly regulates angiogenesis, which is also considered as an important process for carcinogenesis and metastasis [9]. In addition, the overexpression of VEGF has been closely associated with clinical staging, lymph node metastasis, and recurrence of gastric carcinoma [10]. On the other hand, suppression of VEGF signaling has been shown to significantly induce cancer cell death *in vitro* and reduce tumor size *in vivo* [11, 12].

Messenger RNA (mRNA) stability plays a crucial role in VEGF-A post-transcriptional regulation. mRNA decay is an essential step in gene expression to establish the abundance of mRNA in the cytoplasm [13]. The degradation of mRNA is regulated by interactions between specific factors and mRNA structure, including 5’ cap structure, 5’ UTR, 3’ UTR and the 3’ polyadenylate (poly(A)) tail. Further, the most common determinant of RNA stability in mammalian cells is AU-rich elements (ARE) in 3’ UTR [14]. ARE contain multiple copies of the AUUUA motif, which have been shown to regulate the degradation of mRNA through direct or indirect binding with RNA-binding protein [15]. Notably, VEGF-A mRNA stability is reported to be regulated by binding of stabilizing and destabilizing proteins at ARE located in the 3’ UTR region [16]. Numerous RNA-binding protein, AU-rich element RNA binding protein 1 (AUF1), tristetraprolin (TTP), heat shock protein 70 (HSP70), and human antigen R (HuR) were found to bind to VEGF-A mRNA and form ribonucleoprotein complexes that regulate VEGF-A production [17–20]. CRT has been shown to be a novel ARE binding protein that regulates the mRNA degradation of type I angiotensin II receptors and glucose transporter-1 mRNA under high-glucose conditions in several cells [21, 22]. Interestingly, we previously showed that the overexpression of CRT enhanced the expression of RNA binding protein FUBP1, which binds to ARE in 3’ UTR of FUT1 mRNA, and therefore stabilized FUT1 mRNA in J82 bladder cancer cells to promote tumor formation and metastasis [23, 24]. These results suggest that CRT is a trans-acting factor which can regulate mRNA stability. Nevertheless, the mechanism by which CRT regulates VEGF remains unclear. Therefore, we hypothesize that CRT regulates VEGF-A and therefore improves gastric cancer processing and metastasis. To the best of our knowledge, this is the first study to provide evidence of an association between CRT and VEGF-A ARE-determined mRNA stability in gastric cancer.

## Materials and methods

### Patient samples and ethics statement

Written informed consent was obtained from each patient before sample collection. Tissue samples of gastric adenocarcinomas and surrounding non-tumor tissues from 49 patients, who underwent surgery at National Taiwan University Hospital were selected for this study. The study procedures were following governmental regulations (Guidelines for Collection and Use of Human Specimens for Research, Department of Health, Taiwan) and after approval from the Institutional Review Board. The approval number for gastric specimen collection was NO. 201706035RINA from National Taiwan University Hospital, Taiwan. NO. 2013-08-020BC for Human umbilical vein endothelial cell (HUVEC) collection in our previous study was from Taipei Veterans General Hospital, Taiwan [25].

### Cell culture

The human gastric cancer cell line AGS was purchased from Bioresource Collection and Research Center, BCRC (Hsinchu, Taiwan), MKN45 was obtained from the cell line databank in National Taiwan University Hospital (Taipei, Taiwan) [26]. Stable AGS cell lines with CRT overexpression (pEGFP-CRT and pEGFP-C1 as control) and knockdown (CRT-shRNA and shRNA-control) were established as described in our previous study [2]. Briefly, for the construction of EGFP-CRT, full length human CRT was cloned into the pGEM-T easy vector, and then sub-cloned into pEGFP-C1. The following primers were used for the amplification of CRT: BgIII-CRT-F-5′-GAG ATC TAT GCT GCT ATC CGT GCC GC-3′, and BamHI-CRT-R-5′-CCT AGG CTA CAG CTC GTC CTT GGC CTG G-3′. The CRT-shRNA plasmid (TRCN0000019989) and control pLKO.1 vector (TRCN0000072233) were purchased from the National RNAi Core Facility Platform (Academia Sinica, Taipei, Taiwan). The target sequence of CRT-shRNA was 5′-CCA GTA TCT ATG CCT ATG ATA-3′. The cells were maintained under 5% CO_2_ at 37°C in RPMI 1640 (GE Healthcare Life Sciences, Chicago, USA) supplied in 10% fetal bovine serum (Thermo Fisher Scientific, Waltham, Massachusetts, USA) and 1% penicillin/streptomycin (Thermo Fisher Scientific). For the subcultures, the cells were trypsinized using 0.05% EDTA-trypsin (Thermo Fisher Scientific). The Human umbilical vein endothelial cell (HUVEC) was isolated from fresh umbilical cords as previously described [26]. The cell was maintained under 5% CO_2_ at 37°C in Endothelial Cell Growth Medium (Cell Applications, San Diego, California, USA) supplemented with 1% penicillin/streptomycin (Thermo Fisher Scientific). The cell was sub-cultured with 0.05% EDTA-trypsin (Thermo Fisher Scientific) weekly and passaged 2–4 times in the experiments.

### The generation of siRNA transient knockdown cells

To generate transient knockdown MKN45 cell, 20 nM CRT siRNA (sc-29234, Santa Cruz, California, USA) and control siRNA-A (sc-37007, Santa Cruz) were used, followed by transfection with Lipofectamine 2000 (Thermo Fisher Scientific). The cells were harvested after 48 hours of transfection, and further experiments were performed.

### Western blot analysis of tissues and cell lines

Forty-nine pairs of human tumor tissues and adjacent normal tissues were homogenized using RIPA lysis buffer (50 mM Tris, pH 7.4, 1% NP-40, 0.5% Na-deoxycholate, 0.1% SDS, 150 mM NaCl, 2 mM EDTA, 50 mM NaF) supplemented with 1% protease inhibitor Cocktail (Set V, Merck, Darmstadt, Germany). Cell lysates were extracted using 1% NP-40 lysis buffer (20 mM Tris, pH 8.0, 150 mM NaCl, 1% NP-40, 1 mM Na_3_VO_4_, and 10% glycerol) with 1% protease inhibitor Cocktail on ice for 15 minutes, followed by centrifugation at 4°C and 14000 rpm for 15 minutes. The supernatants were then collected and concentrations were measured using a Bradford protein assay (Bio-Rad, Hercules, California, USA) using BSA as a standard. Twenty μg of total protein were denatured at 100°C for 10 minutes and resolved by 10% SDS polyacrylamide gel electrophoresis (PAGE). Proteins were transferred to polyvinylidene difluoride membranes (Merck), which were blocked by 5% BSA in Tris-buffered saline (20 mM Tris, pH 7.4, 150 mM NaCl) containing 0.1% Tween-20 (TBST) for 1 hour before being probed with antibodies overnight at 4°C. The membranes were then washed in TBST and reacted with horseradish peroxidase-conjugated secondary antibodies (Santa Cruz) and developed using a WesternBright ECL detection kit (Advansta, California, USA). The membranes were then imaged using a UVP AutoChemi Image System (Ultra-Violet Products Ltd, California, USA). Primary antibodies to CRT were obtained from Millipore (Darmstadt, Germany) (06–001) and Abcam (Cambridge, UK) (ab2907), and the antibodies to GAPDH (GTX100118) and VEGF-A (GTX102643) were obtained from Genetex (California, USA).

### RNA isolation and quantitative real-time PCR (real-time qPCR)

Total cellular RNA was extracted using TRIzol reagent (Thermo Fisher Scientific). Subsequently, 1 μg of total RNA was reverse transcribed to cDNA using a ReverTra Ace® qPCR RT kit (Toyobo, Osaka, Japan). Real-time qPCR reactions were performed using cDNA (corresponding to 33 ng RNA) with 300 nmol of the primer in SYBR Green Supermix (Bio-Rad) using a Mini-Opticon real-time PCR system (Bio-Rad). The cycle parameters were one denaturation step at 95°C for 15 minutes and 40 cycles of denaturation at 95°C for 30 seconds followed by annealing and elongation at 60°C for 30 seconds, and final elongation at 72°C for 30 seconds. For quantification, the gene expression of each transcript was normalized to GAPDH using the ΔΔCt method. The gene-specific primers were: CRT, forward 5′-AAG TTC TAC GGT GAC GAG GAG-3′, reverse 5′-GTC GAT GTT CTG CTC ATG TTT C-3′; VEGF-A-3’UTR, forward 5′-TGG CAA CTT GTA TTT GTG TGT AT-3′, reverse 5′-ACG GAT AAA CAG TAG CAC CAA TA-3′; and for GAPDH, forward 5′-GGT GGT CTC CTC TGA CTT CAA C-3′, reverse 5′-TCT CTC TTC CTC TTG TGC TCT TG-3′.

### Determination of mRNA stability

To determine the stability of mRNA, 2x10^5^ AGS shRNA-control, CRT-knockdown cells and transient knockdown MKN45 cells were first preincubated with actinomycin D (Act-D) (5 μg/ml) to inhibit transcription and were then collected at 60, 90 and 120 min. Total RNA was subsequently extracted and reverse transcribed as described above. The expression level of VEGF-A mRNA was detected by real-time qPCR and normalized to GAPDH mRNA. The half-life of VEGF-A mRNA was assessed using the one phase decay method of nonlinear regression analysis with GraphPad Prism. Briefly, VEGF-A mRNA level at time 0 (X; 0 minute of the assay) was set as Y_0_. VEGF-A mRNA level at different time points were expressed in the same unit of Y. VEGF-A mRNA level at infinite time was set as the Plateau. Then K is calculated from the equation: Y = (Y_0_ - Plateau)*exp(-K*X) + Plateau. K is the rate constant, expressed in reciprocal of the x-axis time units. Then half-life was computed as ln(2)/K.

### RNA immunoprecipitation

The wild-type, EGFP-C1, and EGFP-CRT AGS cells were lysed in IP lysis buffer (10 mM HEPES, pH 7.0, 100 mM KCl, 5 mM MgCl_2_, 0.5 mM DTT, and 0.5% NP-40) supplemented with 1% protease inhibitor Cocktail (Set V, Merck) and 100 U/ml SUPERase. In^™^ RNase Inhibitor (Thermo Fisher Scientific). The cell lysates were subsequently precleared using non-binding beads, and incubated with the primary antibodies, anti-CRT (ab2907, Abcam), anti-GFP (sc-9996, Santa Cruz), or rabbit and mouse control IgG (sc-2027 and sc-2025, Santa Cruz), at 4°C overnight. This was followed by incubation for 4 hours with either Protein A Mag Sepharose Xtra (GE Healthcare, Chicago, USA) for CRT IP, or Dynabeads M-280 Sheep Anti-mouse IgG (Thermo Fisher Scientific) for GFP IP. The beads were washed and split into two fractions for protein detection and RNA extraction. The immunoprecipitated proteins were eluted from the beads at 95°C, and resolved by 10% SDS-PAGE. The CRT IP membrane was probed with a specific mouse monoclonal CRT antibody (MAB38981, R&D systems, Minnesota, USA), and an anti-GFP antibody (Santa Cruz) was used for the GFP IP membrane. For RNA extraction, the RNA was eluted from the beads in TRIzol reagent (Thermo Fisher Scientific), followed by reverse transcription into cDNA. The target RNA was then analyzed using real-time qPCR. The enrichment of the VEGF-A transcript was normalized to the total amount VEGF-A of the input sample using the ΔΔCt method. The fold changes were calculated relative to the levels of the IgG control.

### Reporter plasmid construction, transfection and dual luciferase activity assay

Synthetic VEGF-A ARE (TCATTTATTTATT) and its mutant (TCAGGTAGGTAGG) were ligated in the C’terminal of *Renilla* luciferase in psiCHECK^TM^-2 between the Not1 and Xhol1 sites to build psiCHECK^TM^-2-VEGF-A ARE and psiCHECK^TM^-2-VEGF-A mARE. For the transfection experiments, AGS cells with CRT overexpression (pEGFP-CRT and pEGFP-C1 as control), and knockdown (CRT-shRNA and shRNA-control) grown to 80% confluence in 12-well plates were transfected with 2 μg of psiCHECK^TM^-2-VEGF-A ARE and psiCHECK^TM^-2-VEGF-A mARE using 5 μl of Lipofectamine TM2000 (Invitrogen, Thermo Fisher Scientific). A Dual-Glo Luciferase reporter assay system (Promega, Fitchburg, Wisconsin, USA) was used to measure the activities of *Renilla* and firefly luciferase at 48 hours post transfection. *Renilla* luciferase was quantified to measure the stability of mRNAs with an inserted ARE at 3’ UTR. In addition, firefly luciferase was measured as an internal control to assess the efficiency of transfection. The results were quantified as the relative ratio of *Renilla* luciferase to firefly luciferase activity.

### Biotinylated RNA probe preparation and Electrophoretic mobility shift assay (EMSA)

Biotinylated ARE containing a partial 3′UTR of the VEGF-A mRNA probe was prepared using *in-vitro* transcription. The ARE sequence from 1755 to 1767 nt of the 3′UTR of the VEGF-A mRNA was used. The nucleotides 1707 through 1817 of the VEGF-A mRNA 3′UTR was first amplified from the Human VEGF-A (NM_001025367) 3’UTR plasmid (SC217125, OriGene, Rockville, USA) using PCR to serve as the wild-type probe sequence. The primer sequences used were: Forward (containing the T7 promoter sequence): 5′-TAA TAC GAC TCA CTA TAG GGA GAA TTC TAC ATA CTA AAT CTC TCT CCT T-3′, Reverse: 5′-GAT GTT AAT ATC TTT TCC CCA CAA T-3′. The ARE deletion probe was established using an overlap extension PCR. The forward primer of wild-type probe and reverse primer carrying the ARE deletion were used to produce the first part of ARE deletion PCR product. Then the reverse primer of wild-type probe and forward primer carrying ARE deletion were used to produce the second part of the ARE deletion PCR product sequence. Next, the first and second part of the ARE deletion PCR product were combined using an overlap extension PCR. Primer sequences used for the ARE deletion were: Forward: 5′-TTG TTA GGT GCT ACT GTT TAT CC-3′, Reverse: 5′-AGC ACC TAA CAA ATA TTA AAA TTA AAA AAG GA-3′. The T7 promoter driven single ARE containing the VEGF-A 3′UTR and the ARE deletion PCR fragment were then transcribed with T7 polymerases (Promega), followed by incubation with Recombinant DNase I (Promega) for 1 hour at 37°C. The RNA probes were then purified by Phenol:Chloroform:Isoamylalcohol (25:24:1), precipitated with ethanol, and then dissolved in DEPC-treated water. After *in-vitro* transcription, the ARE containing the VEGF-A 3′UTR RNA probe was tagged with a biotin-UTP at 3′-end using Pierce™ RNA 3′ End Biotinylation Kit (Thermo Fisher Scientific).

AGS cells were lysed with All Purpose Buffer (50 mM Tris-HCl pH7.5, 250 mM NaCl, 3 mM EDTA, 3 mM EGTA, 1% Triton X-100, 0.5% NP-40, 10% Glycerol) containing 1X protease inhibitor cocktail (Merck). EMSA was used LightShift® Chemiluminescent RNA EMSA Kit (Thermo Fisher Scientific) according to the manufacturer’s instructions. RNA probe at a concentration of 1.25 pmol was heated at 95°C for 5 min and then rapidly cooled down on ice, followed by the addition to 4 μg of cell extract in the absence or presence of 10-fold concentration of unlabeled competitor, 10-fold unlabeled ARE deletion competitor or CRT antibody. 5% non-denaturing polyacrylamide gel containing 0.5X TBE (45 mM Tris-borate, 1 mM EDTA, pH 8.3) was pre-run at 100 V for 1 hour, and the reaction mixtures were then separated on the non-denaturing polyacrylamide gel at 100 V, and transferred to a Nylon membrane at 400 mA. The membrane was crosslinked at 120 mJ/cm^2^ using a UV-light crosslinking instrument equipped with 254 nm bulbs, and the signal was visualized using a Chemiluminescent Nucleic Acid Detection Module (Thermo Fisher Scientific).

### Enzyme-linked immunosorbent assay (ELISA) for VEGF-A

Conditioned media of the AGS shRNA-control and CRT-knockdown cells were collected after 6 and 12 hours, seeded at 5x10^5^ cells/well in 12-well plates, and the expression of VEGF-A was measured using a Human VEGF ELISA kit (Thermo Fisher Scientific). According to the manufacturer’s instructions, 100 μl of conditioned medium and recombinant human VEGF standard were incubated with monoclonal anti-VEGF antibodies for 2 hours at room temperature. The wells were then washed and incubated with detection antibody and streptavidin-HRP, followed by incubation with TMB substrate, and the absorbance was measured using a Microplate reader (Molecular Devices F3, California, USA) at 450 nm after the addition of 100 μl Stop Solution. Data were analyzed using ElisaAnalysis.com (Version 3.2, Leading Technology Group), and a 4-parameter logistic (4PL) curve was fit to the standard curve.

### Endothelial cell proliferation assay in vitro

HUVEC (5x10^3^/well/100 μl medium) were seeded on a 96-well plate with Endothelial Cell Growth Medium (Cell Applications). After 24 hours of culture, the medium was replaced by 100 μl condition medium from AGS shRNA-control or CRT-knockdown cells and treated with 20 ng/ml of VEGF-A recombinant protein (Genetex). At the indicated times, medium containing 50 μl of 5 mg/ml MTT was added to each well for 3 hours. The cells were then gently washed with PBS, followed by the addition of 100 μL of DMSO to dissolve the formazan crystals for 10 minutes. The absorbance was then read at 570 nm. Each experiment was performed in triplicate.

### Statistical analysis

Each result represents at least three independent experiments. Data were statistically analyzed using the t-test or one-way ANOVA (GraphPad Prism, California, USA) in cell and clinical studies. The data are presented as mean ± SD, and a *P* value of < 0.05 was considered to be statistically significant. Overall survival was compared using log-rank comparisons for time-to-event data using the Kaplan-Meier method.

## Results

### CRT was positive correlated with VEGF-A in gastric cancer

**Fig 1:**
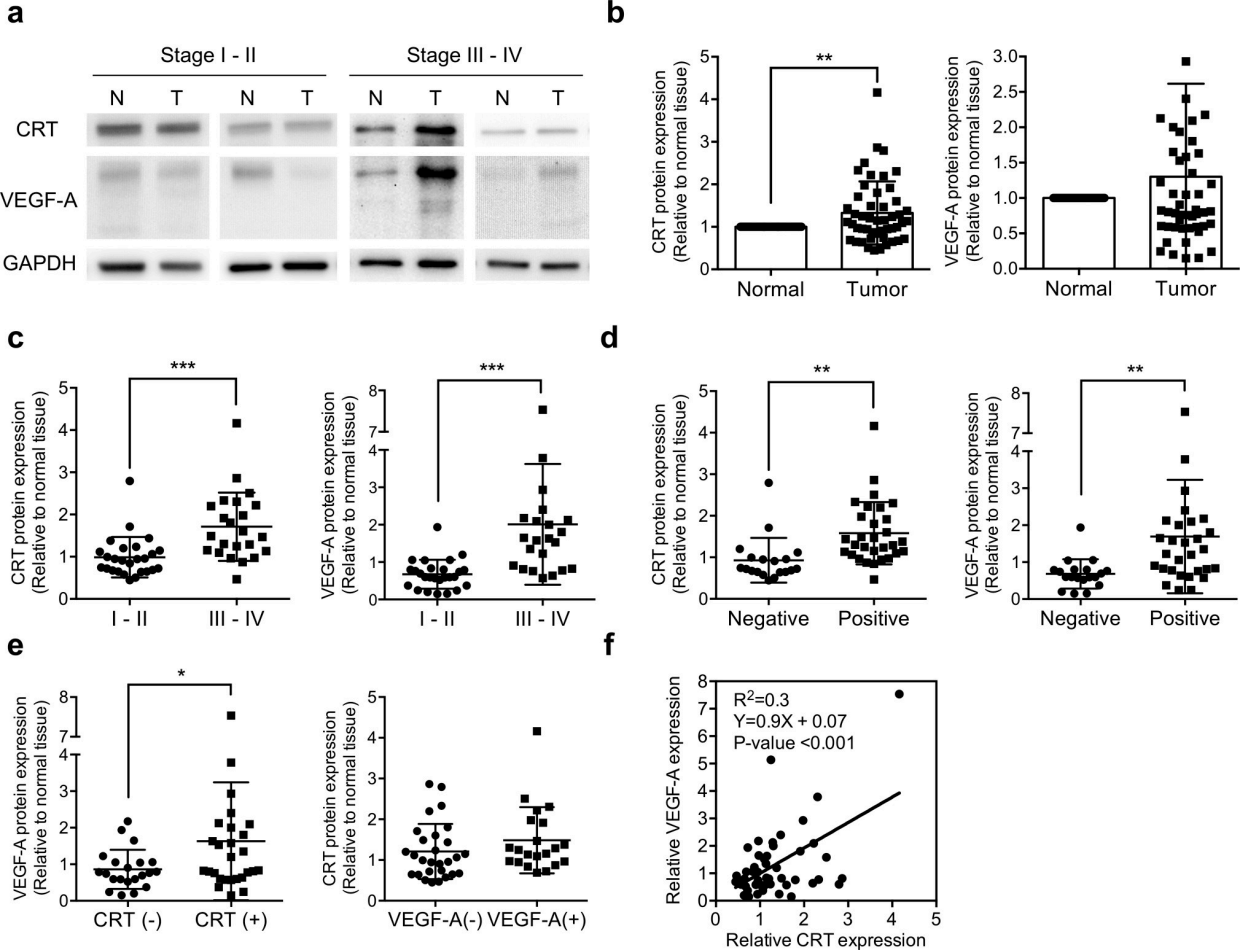
The expression levels of CRT and VEGF-A protein in gastric cancer tissues. Western blot analysis of CRT and VEGF-A from 49 pairs of gastric cancer and matched non-tumor tissues. The expression levels of CRT (63 kD) and VEGF-A (isoform from 27 to 45 kD) were normalized with GAPDH and compared to matched non-tumor tissues. **(a)** Western blot analysis of CRT and VEGF-A from control-tumor pairs of stage I-II and III-IV gastric cancer tissues in different cropped membranes. **(b)** CRT protein expression was significantly higher in the gastric cancer tissues than in adjacent non-tumor tissues, whereas no significant change in VEGF-A was observed in the 49 paired gastric cancer tissues. **(c)** Tumor samples were divided into two groups according to stage for analysis (26 paired stage I-II and 23 paired stage III-IV). Both CRT and VEGF-A protein expressions were higher in the patients with an advanced stage III-IV than in stage I-II. **(d)** Patients were further grouping by nodal status. Both CRT and VEGF-A expressions were higher in 30 paired gastric cancer tissues with positive node metastasis than in 19 node-negative patients. **(e)** Samples were grouped according to CRT (left panel) or VEGF-A (right panel) protein expression, including 21 pairs of a low CRT expression, 28 pairs of a high CRT expression, 28 pairs of a low VEGF-A expression, and 21 pairs of a high VEGF-A expression. VEGF-A protein expression was higher in the CRT overexpressed group, whereas CRT was not altered in the VEGF-A overexpressed group. **(f)** CRT and VEGF-A were positively correlated with gastric cancer. P-values were determined using the t-test (*P ≤ 0.05, **P ≤ 0.01, ***P ≤ 0.001). Error bars represent SD.

To clarify the correlation between CRT and VEGF-A in human gastric cancer, we first examined the protein levels of CRT and VEGF-A in primary gastric cancer tissues and normal gastric tissues adjacent to the tumors from 49 patients. To avoid individual differences, the expression level of CRT and VEGF-A were normalized to GAPDH, and relative to the adjacent normal tissues. The protein levels of CRT and VEGF-A and the clinicopathologic features of the patients with gastric cancer are summarized in Table 1. The enrolled patients included 28 males and 21 females with an age ranging from 37 to 89 years (mean 65 years). The TNM stage was based on the invasion of the gastric wall (T), the involvement of lymph nodes (N), and the presence of distant metastasis (M) [27]. The clinicopathological parameters showed that the expression levels of both CRT and VEGF-A were significantly associated with TNM stage progression and nodal status (N stage) of the patients. In addition, both CRT and VEGF-A were positively correlated with T stage, and VEGF-A was positively significantly correlated with distant metastasis (M stage). However, the expression levels of CRT and VEGF-A were not significantly correlated with gender or age (Table 1). Western blot analysis showed that the expression level of CRT was significantly increased in the 49 gastric cancer tissues compared to the normal tissues (Fig 1B). Further analysis showed that the expression levels of both CRT and VEGF-A were higher in the patients with stage III-IV than stage I-II, and this reflected the strong correlation with clinical stage progression (Fig 1A and 1C). In addition, higher expressions of CRT and VEGF-A were observed in the patients with node metastasis (Fig 1D). VEGF-A was upregulated in the CRT overexpression group, but in contrast CRT was not upregulated in the VEGF-A overexpression group, which suggested a possible CRT-VEGF-A regulation axis (Fig 1E). CRT and VEGF-A were significantly positively correlated (R2 = 0.3, P < 0.001) in the 49 primary gastric cancer tissue samples compared to the adjacent normal tissue samples (Fig 1F).

**Table 1:** CRT and VEGF-A expression levels in relation to clinicopathological features of the gastric cancer patients.

		CRT		VEGF-A	
Characteristics	Total (N)	Expression level^a^	*P*-value	Expression level^a^	*P*-value
Gender					
Male	28	1.33 ± 0.67	0.96	1.12 ± 0.68	0.26
Female	21	1.32 ± 0.8		1.54 ± 1.8	
Age (years)					
<60	18	1.41 ± 0.67	0.53	1.32 ± 0.88	0.94
≥60	31	1.28 ± 0.77		1.29 ± 1.49	
TNM stage					
I + II	26	0.99 ± 0.47	0.0003 ***	0.78 ± 0.38	0.0002 ***
III + IV	23	1.71 ± 0.79		2.01 ± 1.58	
T stage					
I + II	19	1.07 ± 0.66	0.05 *	0.77 ± 0.39	0.02 *
III + IV	30	1.49 ± 0.73		1.64 ± 1.54	
Nodal status					
Negative	19	0.93 ±0.36	0.002 **	0.68 ± 0.39	0.007 **
Positive	30	1.58 ± 0.74		1.69 ± 1.5	
Metastasis					
Negative	43	1.27 ± 0.62	0.14	0.994 ± 0.65	0.0001 ***
Positive	6	1.7 ± 1.21		3.5 ± 2.29	

Data represented mean± SD. *P*-value were determined by t-test.

*p≤ 0.05

**p≤ 0.01

***p≤ 0.001)

^a^ The expression level was normalized to GAPDH and presented as a fold change compared to matched non-tumor tissues.

**Fig 2:**
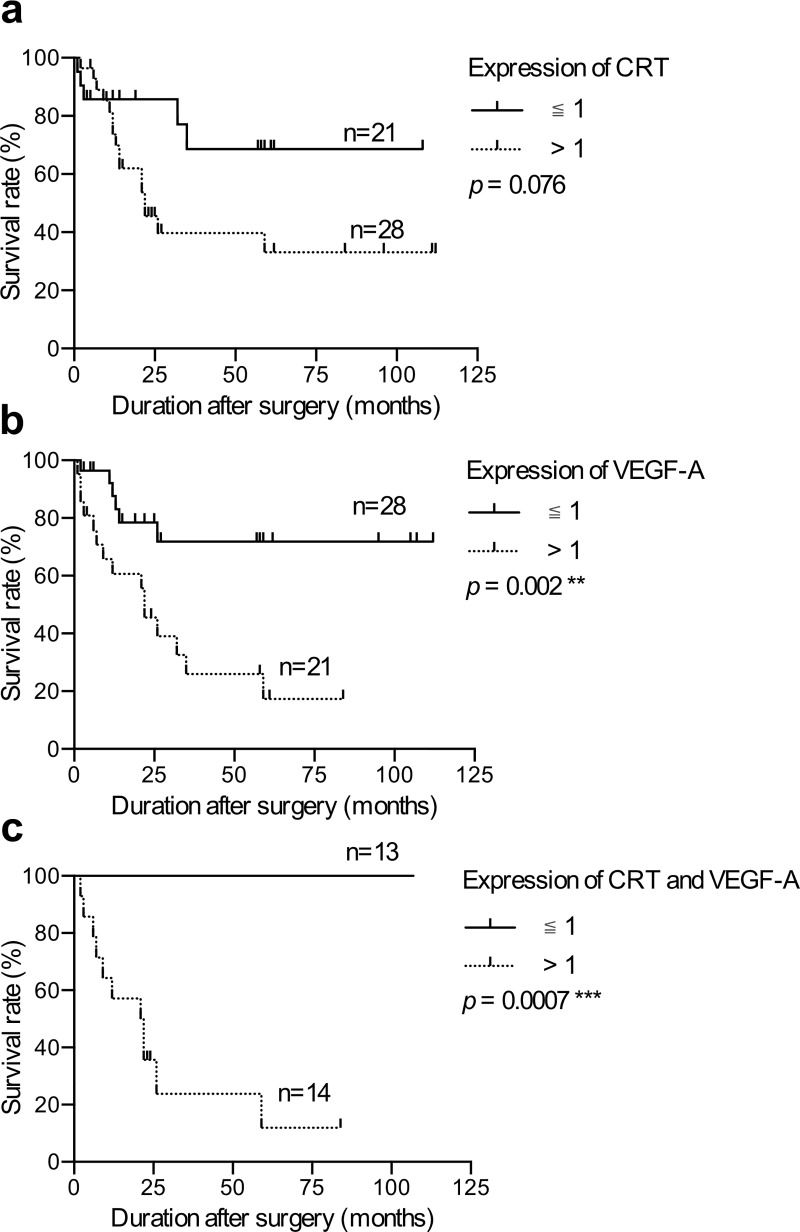
Survival analysis of the patients with gastric cancer. Kaplan-Meier survival analysis according to the expression of CRT and VEGF-A in the gastric cancer patients. The patients were plotted with the expression of CRT and VEGF-A normalized to GAPDH in Western blot and grouped according to the expression level (>1 and ≤1). **(a)** CRT expression was not significantly associated with survival of the 49 gastric cancer patients. **(b)** VEGF-A expression was significantly associated with survival of the 49 gastric cancer patients. **(c)** 14 patients with a high expression of both CRT and VEGF-A had poor survival. P-values were determined using the log-rank test (**P ≤ 0.01, ***P ≤ 0.001).

The patients were further grouped according to the protein level of CRT or VEGF-A, and Kaplan-Meier analysis was performed to determine the correlations between CRT and VEGF-A and survival. Of the 49 gastric cancer patients in this study, the expression of CRT was not significantly associated with survival (P = 0.076, Fig 2A), whereas the patients with a high level of VEGF-A were associated with poor survival (P = 0.002, Fig 2B). Moreover, the patients with co-overexpressions of CRT and VEGF-A had the worst prognosis compared to the other patients (P = 0.0007, Fig 2C). These results clearly indicated that CRT and VEGF-A were positively correlated, and both played a crucial role in the progression of gastric cancer. Moreover, the co-overexpression of CRT and VEGF-A in gastric tumors further contributed to the poor survival of the patients.

### CRT induced the expression of VEGF-A by regulating its mRNA stability

**Fig 3:**
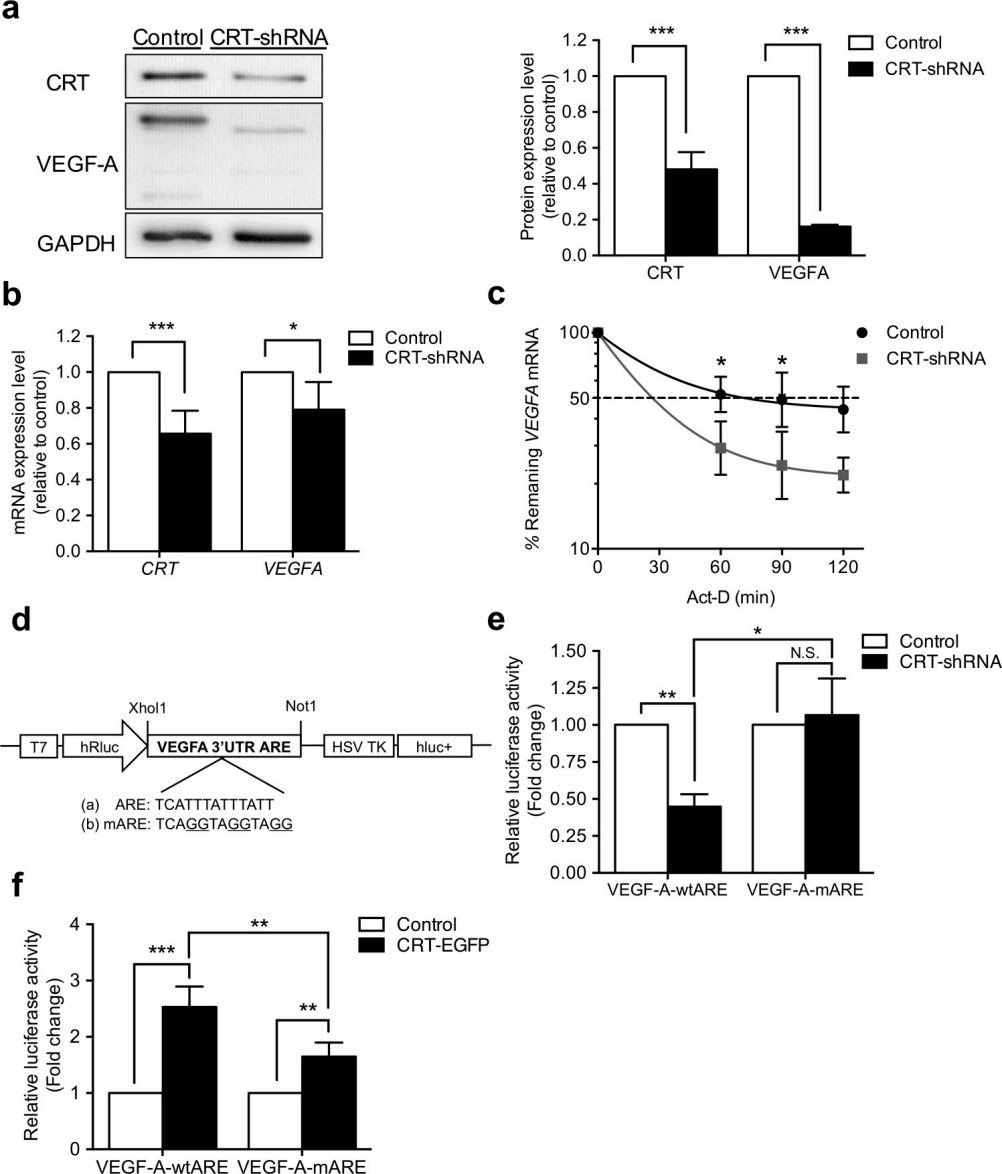
Knockdown of CRT suppressed VEGF-A expression and mRNA stability through ARE in AGS cells. AGS cells were transfected with control or CRT-shRNA plasmids to generate CRT stable knockdown cells. (a) Western blot analysis demonstrated protein levels of CRT and VEGF-A in AGS stable cells. Human GAPDH was used as a loading control. Significant reductions of CRT and VEGF-A protein expressions were observed in CRT knockdown cells. (b) mRNA expressions of CRT and VEGF-A-3’ UTR were analyzed by real-time PCR in AGS stable cells. The expression level of GAPDH was used for normalization as the internal control. Both CRT and VEGF-A mRNA expressions were inhibited in the CRT knockdown cells. (c) Cells were treated with 5 μg/ml actinomycin-D, and total RNA was harvested at the four indicated time points (0, 60, 90, 120 minutes). The amounts of VEGF-A mRNA of the shRNA-control and CRT-knockdown cells were quantified by real-time PCR. VEGF-A mRNA was significantly less stable in the CRT-knockdown cells with lower half-life and 50% mRNA remaining time. The remaining amount of VEGF-A mRNA in the CRT-knockdown cells was significantly decreased at 60 and 90 minutes compared to that in the control cells. (d) Schematic diagram of reporter constructs containing T7-driven *Renilla* luciferase (hRluc) fused to wild-type or mutant ARE sequences and HSV TK-driven firefly luciferase (hluc+) as the internal control. (e) shRNA-control and CRT-knockdown AGS cells were transfected with the plasmids shown in panel d. A Dual-Glo Luciferase reporter assay system was used to measure the activity of *Renilla* and firefly luciferase after 48 hours transfection. The ratio of *Renilla* luciferase activity and firefly luciferase was quantified. The ratio of *Renilla*/firefly luciferase activity was significantly reduced with VEGF-A-wtARE in the CRT-knockdown cell, whereas this perturbation was abolished in VEGF-A-mARE cells. (f) EGFP-CRT and EGFP-C1 AGS cells were transfected with the plasmids shown in panel d. The ratio of Renilla luciferase activity and firefly luciferase was quantified. The ratio of Renilla/firefly luciferase activity was significantly increased from VEGF-A-wtARE in EGFP-CRT cell, and the ratio was higher than VEGF-A-mARE transfected cell. Data are presented as mean± SD for three independent experiments. *P*-values were determined using the t-test and two way ANOVA (*P ≤ 0.05, **P ≤ 0.01, ***P ≤ 0.001).

CRT has been demonstrated to regulate FUT 1 expression by sustaining its mRNA stability [24]. Further, the ARE of VEGF-A mRNA has been shown to contribute to mRNA stability through interactions with RNA binding proteins such as Hsp70 and HuR [19, 28]. This prompted us to investigate whether CRT regulated the expression of VEGF-A by affecting mRNA stability. To verify this post-transcriptional mechanism, CRT stable knockdown AGS cells [2] were generated by CRT-shRNA transfection and siRNA knockdown on MKN45 cells. Results showing marked down-regulation of VEGF-A in both cell lines. Real-time qPCR and Western blot results showed that CRT down-regulation attenuated the expression levels of VEGF-A protein and mRNA (Fig 3A and 3B, S1A Fig). Consistent with the results from the patients’ gastric cancer samples (Fig 1), the expression level of VEGF-A was governed by the level of CRT. To calculate the kinetic decay of the VEGF-A mRNA, actinomycin-D (Act-D) was used to inhibit the transcription of the mRNA. AGS and MKN45 cells were incubated with Act-D between 60–120 min, and the mRNA was collected at the indicated time points. The half-life of the endogenous VEGF-A mRNA was less stable (23%) following CRT knockdown in the AGS cells (t_1/2_~ 18 min, Fig 3C) than in the control cells (t_1/2_~ 23 min, Fig 3C). The time of 50% mRNA remaining was also calculated. The 50% VEGF-A remaining was almost 60% less stable in the CRT knockdown cell (~26 min, Fig 3C) than in the control AGS cell (~72 min, Fig 3C). The influence of CRT knockdown on VEGF-A stability was also observed in MKN45 cell with CRT siRNA knockdown (S1B Fig). These results strongly suggest that CRT affects the mRNA level and stability of VEGF-A.

### CRT stabilized VEGF-A mRNA through ARE region

Previous studies have indicated that ARE, adenosine and uridine nucleotide rich specific sequences in 3’ UTR, are the major elements in controlling mRNA stability [29–31]. The stability of VEGF-A is also reported to be mainly mediated through 3′UTR, which contains several AREs [16, 32]. In this study, the class II ARE located near the end of the 3′UTR, which is the only ARE carrying two copies the consensus ARE sequence (AUUUA) was chosen based on previous publications and an online database (King Faisal Specialist Hospital & Research Center, Riyadh, Saudi Arabia). These ARE regions are further targeted by ARE-binding proteins to stabilize or destabilize the target mRNA. To investigate whether CRT controls VEGF-A mRNA stability by regulating the ARE in the 3′UTR of VEGF-A, wild-type (TCATTTATTTATT) or mutant VEGF-A ARE sequences (TCAGGTAGGTAGG) were inserted into the downstream sequence of the Renilla luciferase coding sequence in a psiCHECK-2 vector to create VEGF-A-wtARE and VEGF-A-mARE luciferase reporters (Fig 3D). The Renilla luciferase activity was then monitored in CRT-knockdown, CRT overexpression (EGFP-CRT), and each control AGS cells. The readings were then normalized to Firefly luciferase as an internal control. The ratio of Renilla/firefly luciferase activity from the VEGF-A-wtARE, but not the VEGF-A-mARE luciferase reporter was reduced approximately 55% in the CRT-knockdown cells (Fig 3E). Furthermore, EGFP-CRT overexpression cells were used to confirm the CRT regulation of VEGF-A ARE (S2 Fig). The luciferase activity was increased in VEGF-A-wtARE transfected EGFP-CRT cells, and the ratio was significantly higher than for VEGF-A-mARE transfected cells (Fig 3F). These results demonstrated that CRT controls VEGF-A mRNA stability through VEGF-A ARE regions.

### CRT is involved in VEGF-A RNA binding protein complexes

**Fig 4:**
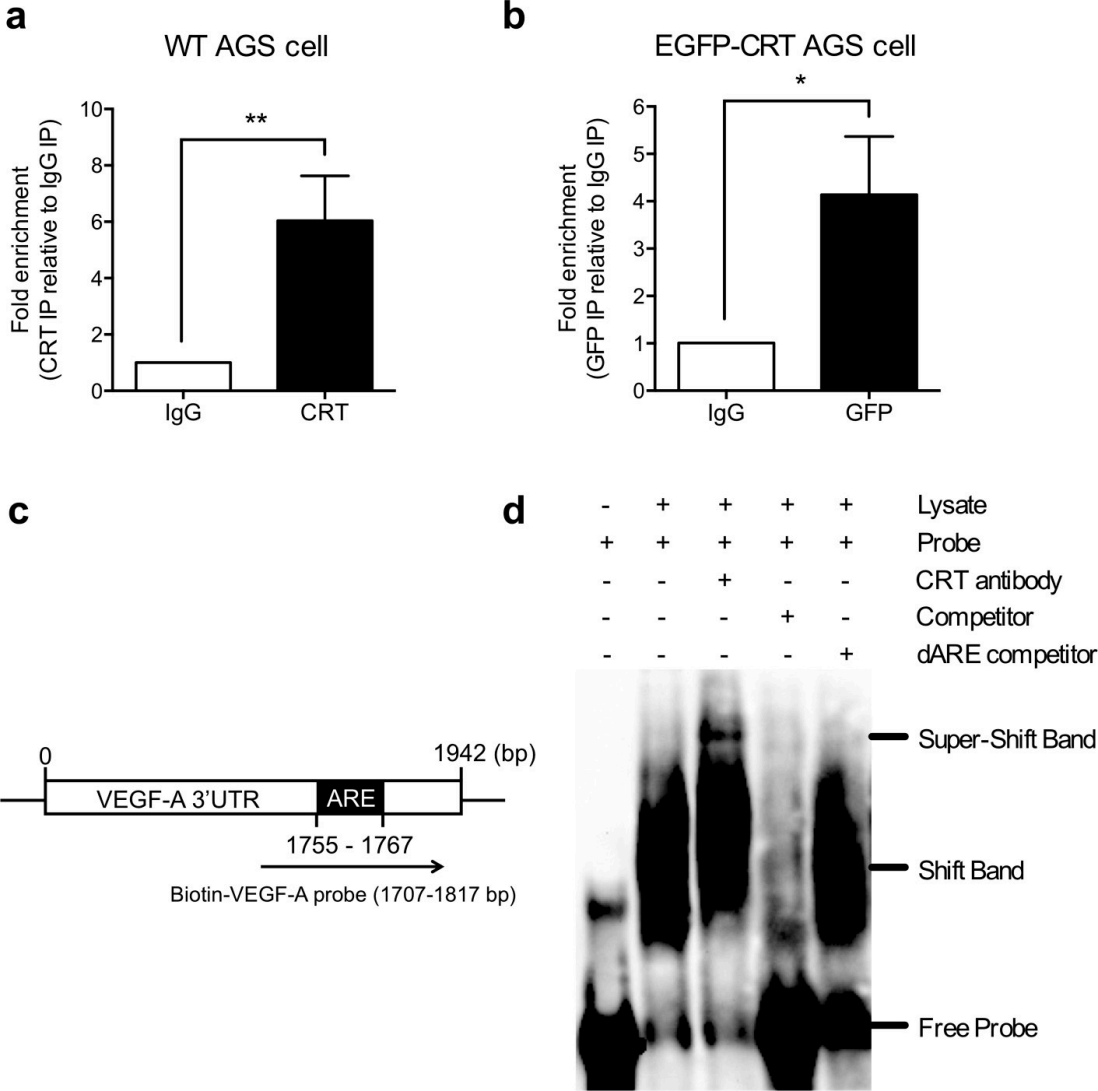
CRT was a *VEGF-A* mRNA-binding protein. (a) AGS cell lysates were incubated with CRT or control IgG antibodies, followed by RNA immunoprecipitation (RNA-IP). Total RNA was extracted from the pull-down complexes and subjected to real-time qPCR with the primers specific to *VEGF-A*. The enrichment of VEGF-A mRNA was normalized to the total amount VEGF-A in the input lysate, and then compared to the levels in the IgG control. An enrichment of ~6-fold is shown. (b) EGFP-tagged CRT cell lysates were incubated with GFP or control IgG antibodies, followed by RNA immunoprecipitation (RNA-IP). Real-time qPCR results showed a 4-fold enrichment of EGFP-CRT binding to VEGF-A mRNA from GFP IP compared to the control IgG IP. (c) 1.25 pmol biotin labeled ARE containing VEGF-A RNA probe was incubated with AGS protein lysate in the absence or presence of 10-fold unlabeled probe (competitor), 10-fold unlabeled ARE deletion probe (dARE competitor) or CRT antibody. The shift band was indicated a specific RNA-protein complex and super-shift band was RNA-target protein-antibody complex. All experiments were repeated at least three times. Histograms represent mean± SD for three independent experiments. *P*-values were determined using the t-test (*P ≤ 0.05).

To further investigate the post-transcriptional regulation of VEGF-A expression, RNA immunoprecipitation and electrophoretic mobility shift assay (EMSA) were performed to assess the interaction between CRT and VEGF-A mRNA. Total lysates of AGS cells were pulled-downed by the specific CRT antibody and IgG control for RNA immunoprecipitation. The presence of CRT in anti-CRT pull-down complexes was confirmed by Western blot (S3A Fig). The RNA fractions were extracted from the CRT IP, IgG control IP, and input lysates and verified by real-time qPCR to measure whether the endogenous VEGF-A mRNA was pulled down by CRT IP. VEGF-A mRNA from the CRT IP was normalized to the total VEGF-A amount of the input lysate, and then compared to the level from the IgG control. Real-time qPCR results revealed an approximate 6-fold enrichment in VEGF-A mRNA from CRT IP compared to the IgG control IP (Fig 4A). To further confirm the RNA binding results from the endogenous CRT pull-down, lysates from the EGFP-CRT overexpression AGS cells were used for IP using either an anti-GFP or control IgG antibody (S3B Fig). The binding of EGFP-CRT to VEGF-A mRNA showed 4-fold enrichment in the GFP IP than that in the control IgG IP (Fig 4B). This abundance was specific in the EGFP-CRT overexpression cells, but not in the EGFP-C1 control cells (S3C Fig). These results suggested that CRT interacted with endogenous VEGF-A mRNA in AGS cells.

Moreover, EMSA was further performed to validate the ARE binding activity of CRT to VEGF-A mRNA. To generate an RNA probe for the detection, a T7 promoter driven single ARE containing the VEGF-A 3′UTR fragment was amplified by PCR, then *in vitro* transcribed with T7 polymerase (Fig 4C). A 111 nt biotin-labeled ARE containing the VEGF-A RNA probe was incubated with AGS protein lysate in the absence or presence of 10-fold of unlabeled probe (competitor), 10-fold of unlabeled ARE deletion probe (dARE competitor) or CRT antibody. An upward shift in the mobility of VEGF-A ARE containing RNA probe was shown for protein concentration up to 4 μg (Fig 4D, S4A Fig). 1- to 10-fold competitor with the same sequence (biotin-unlabeled probe) was added to compete with the binding, thereby verifying the specificity of the probe interaction (S4B Fig). Most of all, a super-shift band appeared when the RNA probe was incubated with both the lysate and the anti-CRT antibody (Fig 4D), suggesting that CRT was an VEGF-A RNA-binding protein. Further investigation using excess amounts of ARE deletion biotin-unlabeled probe (dARE competitor) showed no competitive binding, indicating the ARE-specific binding activity of CRT (Fig 4D). These findings demonstrate that the binding of CRT to the VEGF-A 3′UTR is dependent on the ARE element, suggesting that CRT forms an mRNA-protein binding complex on the ARE of the VEGF-A mRNA, and consequently affects the mRNA stability of VEGF-A.

### CRT regulated endothelial cell proliferation through the VEGF-A pathway

During cancer progression, angiogenesis plays an essential role in supporting tumor growth and proliferation by supplying oxygen and nourishment, and further stimulating tumor migration and invasion [33]. In the physiological environment of most solid tumors, cancer cells secrete cytokines and angiogenic molecules to promote angiogenesis and facilitate their invasion. VEGF-A has been reported to be a major inducer of vascular permeability and a potent angiogenic cytokine for tumor metastasis. Our findings indicated that CRT regulated VEGF-A ARE regions to stabilize VEGF-A mRNA in AGS cells. To elucidate whether this CRT-VEGF-A axis mechanism in AGS cells affected angiogenesis of endothelial cells, conditioned media from shRNA-control or CRT-knockdown AGS cells were transferred to HUVECs to mimic the microenvironment that triggers tumor angiogenesis.

**Fig 5:**
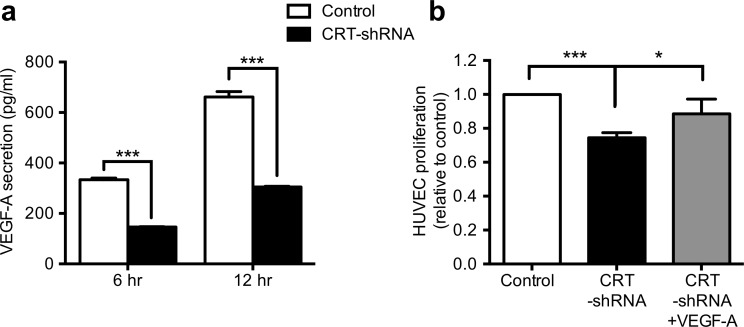
CRT regulated endothelial cell proliferation via the VEGF-A signaling pathway. (a) ELISA was performed to analyze the concentration of VEGF-A in conditioned medium of shRNA-control or CRT-knockdown AGS cells. After 6 to 12 hours culture, the concentration of VEGF-A was significantly decreased in CRT-knockdown cells. (b) HUVECs were further treated with conditioned medium collected from shRNA-control or CRT- knockdown cells, and 20 ng/ml exogenous VEGF-A recombinant protein was added to the CRT-knockdown cells to investigate the VEGF-A signaling pathway. After incubation for 24 hours, the proliferation of HUVECs was analyzed by MTT assays. The proliferation rate was significantly reduced in the CRT-knockdown group, and rescued by the addition of VEGF-A. Each histogram represents quantified results as the mean ± SD. *P*-values were determined using the t-test or one-way ANOVA (*P ≤ 0.05, ** *P* ≤ 0.001).

The concentration of VEGF-A protein was first analyzed by ELISA. To avoid the FBS background, the mean concentrations of VEGF-A protein secreted from the AGS cells into serum free medium after 12 hours of incubation were 661.8 pg/ml for shRNA-control AGS cells and 305.1 pg/ml for CRT knockdown cells, reflecting a significant perturbation of VEGF-A synthesis and secretion upon reduction in CRT (Fig 5A). The conditioned medium was then transferred to HUVECs for 24 hours incubation, and the cell proliferation rate of the HUVECs was analyzed using MTT assays. Compared to the conditioned medium from the AGS shRNA-control cells, the proliferation rate of HUVECs with conditioned medium from the CRT knockdown cells was severely inhibited (Fig 5B). To determine whether VEGF-A was responsible for the stimulation of HUVEC proliferation, 20 ng/ml of VEGF-A recombinant protein was added to the conditioned medium. Reduced cell proliferation of HUVECs with conditioned medium from CRT knockdown cells was rescued by the addition of exogenous VEGF-A recombinant protein (Fig 5B). In conclusion, our results suggested that CRT maintained VEGF-A mRNA stability by interacting with its ARE. Consequently, the gradual increase in CRT during tumor development induces VEGF-A secretion and further promotes angiogenesis in aggressive gastric cancer.

## Discussion

CRT has been reported to affect the progression of gastric cancer patients, and also regulate VEGF-A expression in several cancer cell lines. However, the correlation of CRT and VEGF-A in gastric cancer clinical research and the mechanism by which CRT regulates VEGF remain unrevealed. In this study, we first demonstrated that CRT was positively correlated with VEGF-A in gastric cancer tissue samples. Further, expression levels of CRT and VEGF-A were both overexpressed during tumor stage progression (TNM stage) and nodal metastasis in patients with gastric cancer. Of note, the expression of VEGF-A was higher in the CRT overexpressed group, suggesting a possible CRT-VEGF-A regulation axis. Moreover, we found that the patients with high levels of both CRT and VEGF-A had a worse prognosis. These findings suggest that the co-expression of CRT and VEGF-A plays a crucial role in the progression of gastric cancer. Thus, it is imperative to clarify the underlying regulatory mechanism of CRT and VEGF-A.

CRT is best known as an ER chaperon protein with well-defined protein folding properties in translational regulation to increase the yield of correctly folded proteins, and their assembly [34]. On the other hand, the formation and secretion of VEGF-A, a secreted proteins, is orchestrated by various chaperones, including the oxygen-regulated protein (ORP) 150 and αB-crystallin/HSPB5 [35, 36]. Therefore, it is possible that CRT post-translationally regulates VEGF-A. However, the association of CRT with the VEGF-A mRNA has not been determined. This study was aimed at understanding the function of CRT in the regulation of VEGF-A mRNA stability. VEGF-A mRNA stability is reported to be regulated by at least five ARE sequences located within the 3’ UTR region [16, 32]. The only ARE carrying two copies the consensus ARE sequence (AUUUA) was chosen in this study. We provide the evidence for a novel regulatory role of CRT in VEGF-A mRNA stability in a 3′UTR ARE element-dependent manner. Predictably, other ARE sequences within the 3’UTR also play the same role in this regulation. On the other hand, the inhibitory effect of CRT on VEGF-A mRNA stability is not statically significant after 120 min. We therefore evaluated the expression of Heat shock protein 70 (HSP70), which is also a VEGF-A RNA ARE binding regulator to stabilize VEGF-A mRNA. The result showed that CRT knockdown has no affect the expression of HSP70 (S5 Fig). We propose that other VEGF-A mRNA regulators in CRT knockdown cell maintaining the mRNA stability at some level. On the other hand, Act-D is a strong transcription inhibitor and the VEGF-A mRNA is very unstable and easily degraded. As a result, no significant difference of VEGF-A mRNA between control and knockdown group was observed after a long time treatment. Notably, CRT knockdown caused a strong reduction (almost 80%) in VEGF-A expression compared to control cells. This strongly suggests that CRT is a dominant regulator on VEGF-A. To further determine whether CRT stabilizes VEGF-A mRNA through ARE sequences, ARE mutant VEGF-A 3’ UTR was constructed. The mutation in the VEGF-A ARE specifically abolished the influence of CRT on the target gene in both CRT knockdown and overexpressing cells, suggesting that CRT stabilizes the mRNA stability of VEGF-A through ARE. In addition, these results strongly consistent with the positive correlation of CRT and VEGF-A in the clinical results. Most importantly, we also revealed the involvement of CRT in the VEGF-A mRNA ARE-binding protein complex through multiple assays. During tumorigenesis, the release of VEGF-A has been demonstrated to stimulate the growth of new blood vessels from nearby capillaries to support the lack of oxygen and nutrients for tumor cells [37–39]. Of note, we confirmed that secreted VEGF-A in conditioned medium was disturbed in CRT knockdown AGS cells. Consequently, the conditioned medium from CRT knockdown AGS cells did not stimulate HUVEC proliferation to the same extent as intact AGS cells. These results strongly suggest that the presence of CRT ensures the stable production of VEGF-A as an angiogenesis factor by binding to VEGF-A ARE.

In our previous study on bladder cancer, we showed that CRT was not part of the ARE binding protein complex and that it indirectly stabilized FUT1 mRNA degradation through activating the ARE binding protein FUBP1. This is in contrast to our latest findings in which CRT was present in the VEGF-A mRNA binding complex to stabilize VEGF-A mRNA. Various studies have suggested that CRT may act as an RNA binding protein to regulate target gene translation, such as rubella virus RNA, GCN repeats within C/EBPα and C/EBPβ and p21 mRNA [40–42]. CRT has also been shown to have ribonucleoprotein binding domains to bind RNA [40]. Furthermore, CRT has been reported to be an ARE binding protein in several cells [21, 22]. A possible explanation for the contradiction between our two studies is that CRT may regulate RNA stability of different genes through distinct mechanisms. Nevertheless, further studies are needed to elucidate whether CRT directly interacts with VEGF-A ARE or interacts with VEGF-A ARE binding proteins to stabilize mRNA. For instance, TTP and HSP70 are known VEGF-A ARE binding proteins which bind to the mRNA and form ribonucleoprotein complexes that regulate VEGF-A production. HSP70 have been demonstrated to increase VEGF-A stability, whereas TTP acts as a negative regulator of VEGF-A gene expression [17, 18, 29]. We speculate that CRT may possess a competitive or cooperative interaction with these regulators to manipulate VEGF-A stability. The detailed mechanism of the binding is an interesting area that needs to be investigated in future studies.

In conclusion, the present study is the first to verify that CRT positively correlated to VEGF-A in clinical progression, and involves in the VEGF-A RNA binding protein complex to act as an mRNA stabilizing mediator. In addition, the axis of CRT-ARE-VEGF machinery promotes secretion of VEGF-A and functions to increase endothelial cell proliferation. CRT has also been reported to be overexpressed in multiple cancers including breast cancer, hepatocellular carcinoma, pancreatic cancer, leukemia and esophageal cancer as well as gastric cancer [43–47]. Moreover, CRT has been shown to be a crucial regulator of VEGF-A expression, angiogenesis and metastasis in some of these cancers [2, 4, 6, 7, 48]. The novel findings of this study may be useful in future research on other cancers highly expressing CRT and VEGF-A. Our findings may contribute to elucidate the mechanisms of the progression and metastasis of these cancers to provide a potential therapeutic target in anti-cancer therapy.

## Supporting information

S1 Fig Knockdown of CRT suppressed VEGF-A expression in MKN45 cells. MKN45 cells were transfected with control or CRT-siRNA to generate CRT knockdown cells. (a) Western blot analysis demonstrated the protein level of CRT and VEGF-A in the MKN45 cells. Human GAPDH was used as a loading control. A significant reduction in CRT and VEGF-A protein expressions were observed in the CRT knockdown cells. (b) Cells were further treated with 2.5 μg/ml actinomycin-D, and total RNA was harvested at the four indicated time points (0, 60, 90, 120 minutes). The amounts of VEGF-A mRNA of the control and CRT-knockdown cells were quantified by real-time PCR. VEGF-A mRNA was significantly less stable in the CRT-knockdown cell (t1/2 = 10.35 min) than in the control cell (t1/2> 120 min). The amount of VEGF-A mRNA was significantly decreased after 60 and 90 minutes. P-values were determined using ANOVA (*P ≤ 0.05, **P ≤ 0.001).
(TIFF)

S2 Fig Western blot analysis in EGFP-CRT AGS cells. AGS cells were transfected with pEGFP-CRT or pEGFP-C1 control to generate CRT overexpression AGS cell. Western blot analysis demonstrated the endogenous CRT (63 kD), overexpressed EGFP (27kD), and EGFP-CRT (90kD). Data are presented as mean± SD for three independent experiments.
(TIFF)

S3 Fig The pulled-down complexes from CRT and EGFP IP. (a) AGS cell lysate was immunoprecipitated (IP) with either CRT or IgG control antibodies, followed by immunoblotting (IB) with anti-CRT antibodies to evaluate the pull-down specificity. (b) EGFP-CRT AGS cell lysate was IP with either GFP or IgG control antibodies. Western blot analysis demonstrated the pull-down specificity of overexpressed EGFP-CRT (90 kD). (c) EGFP-C1 control AGS cell lysate was incubated with either GFP or IgG control antibodies, followed by RNAIP. The enrichment of VEGF-A mRNA was normalized to the total amount VEGF-A of input and then compared to the levels in the IgG control. Real-time qPCR showed no significant change from GFP IP than control IgG IP.
(TIFF)

S4 Fig Concentration of AGS protein lysate and WT ARE competitor with EMSA analysis. (a) Biotin labeled RNA probe was incubated with different concentration of AGS protein lysate (form 1 to 4 μg). The shift band was indicated a specific RNA-protein complex. (b) 1.25 pmol biotin labeled ARE containing VEGF-A RNA probe was incubated with AGS protein lysate in the absence or presence of 1-10-fold unlabeled probe (same sequence competitor).
(TIFF)

S5 Fig Knockdown of CRT had no effect on HSP70 expression in MKN45 cells. MKN45 cells were transfected with control or CRT-siRNA to generate CRT knockdown cells. Western blot analysis demonstrated the protein level of CRT and HSP70 in the MKN45 cells. Human GAPDH was used as a loading control.
(TIFF)

S6 Fig Full-length membranes and gels. (PDF)
