# Supplementary figures and images for: Calreticulin regulates vascular endothelial growth factor-A mRNA stability in gastric cancer cells
Source: PLoS One. 2019 Nov 14;14(11):e0225107. doi: 10.1371/journal.pone.0225107 (PMC6855450; doi:10.1371/journal.pone.0225107)

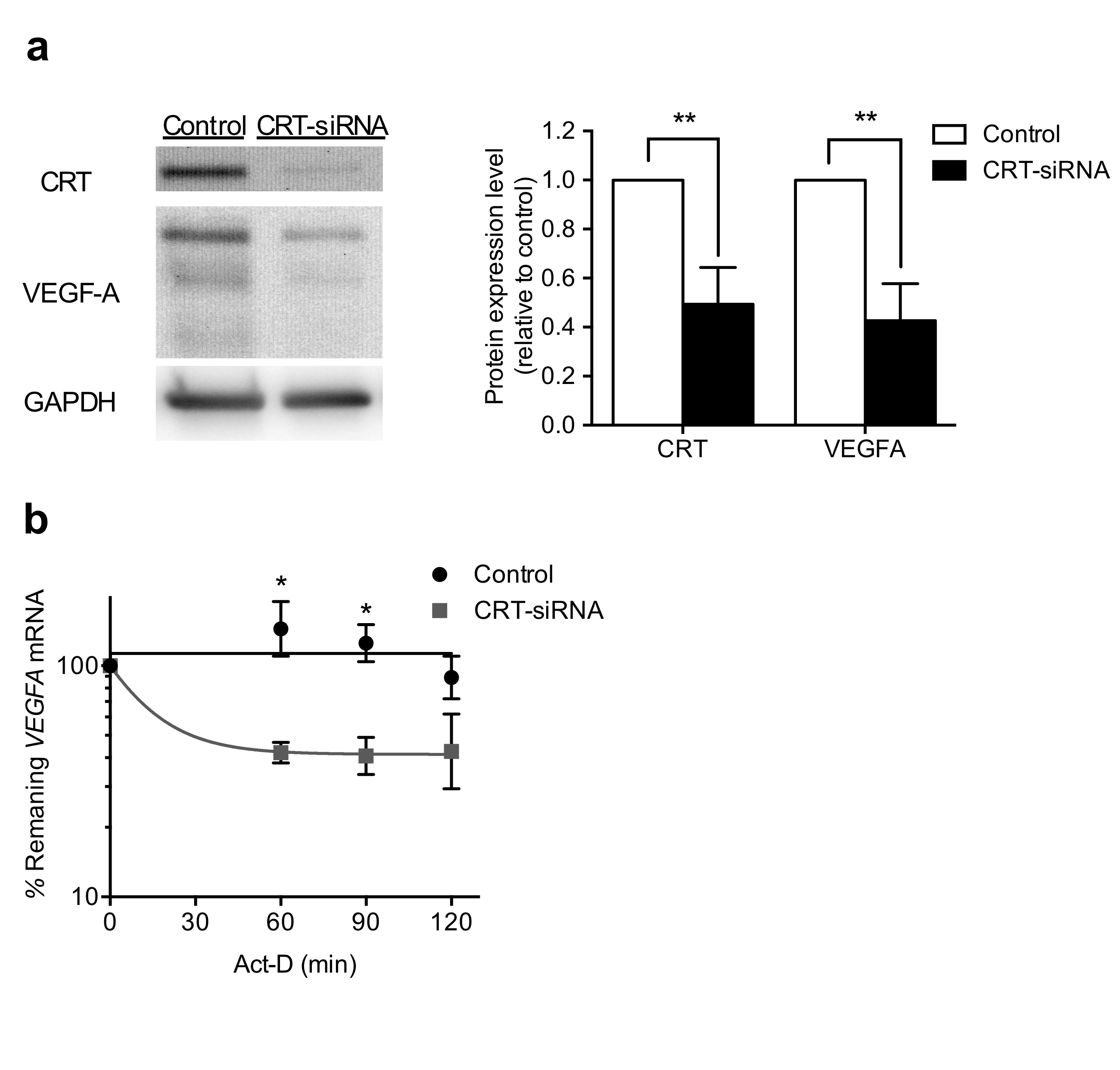

Supplement: S1 Fig — MKN45 cells were transfected with control or CRT-siRNA to generate CRT knockdown cells. (a) Western blot analysis demonstrated the protein level of CRT and VEGF-A in the MKN45 cells. Human GAPDH was used as a loading control. A significant reduction in CRT and VEGF-A protein expressions were observed in the CRT knockdown cells. (b) Cells were further treated with 2.5 μg/ml actinomycin-D, and total RNA was harvested at the four indicated time points (0, 60, 90, 120 minutes). The amounts of VEGF-A mRNA of the control and CRT-knockdown cells were quantified by real-time PCR. VEGF-A mRNA was significantly less stable in the CRT-knockdown cell (t1/2 = 10.35 min) than in the control cell (t1/2> 120 min). The amount of VEGF-A mRNA was significantly decreased after 60 and 90 minutes. P-values were determined using ANOVA (*P ≤ 0.05, **P ≤ 0.001). (TIFF) [file pone.0225107.s001.tiff]

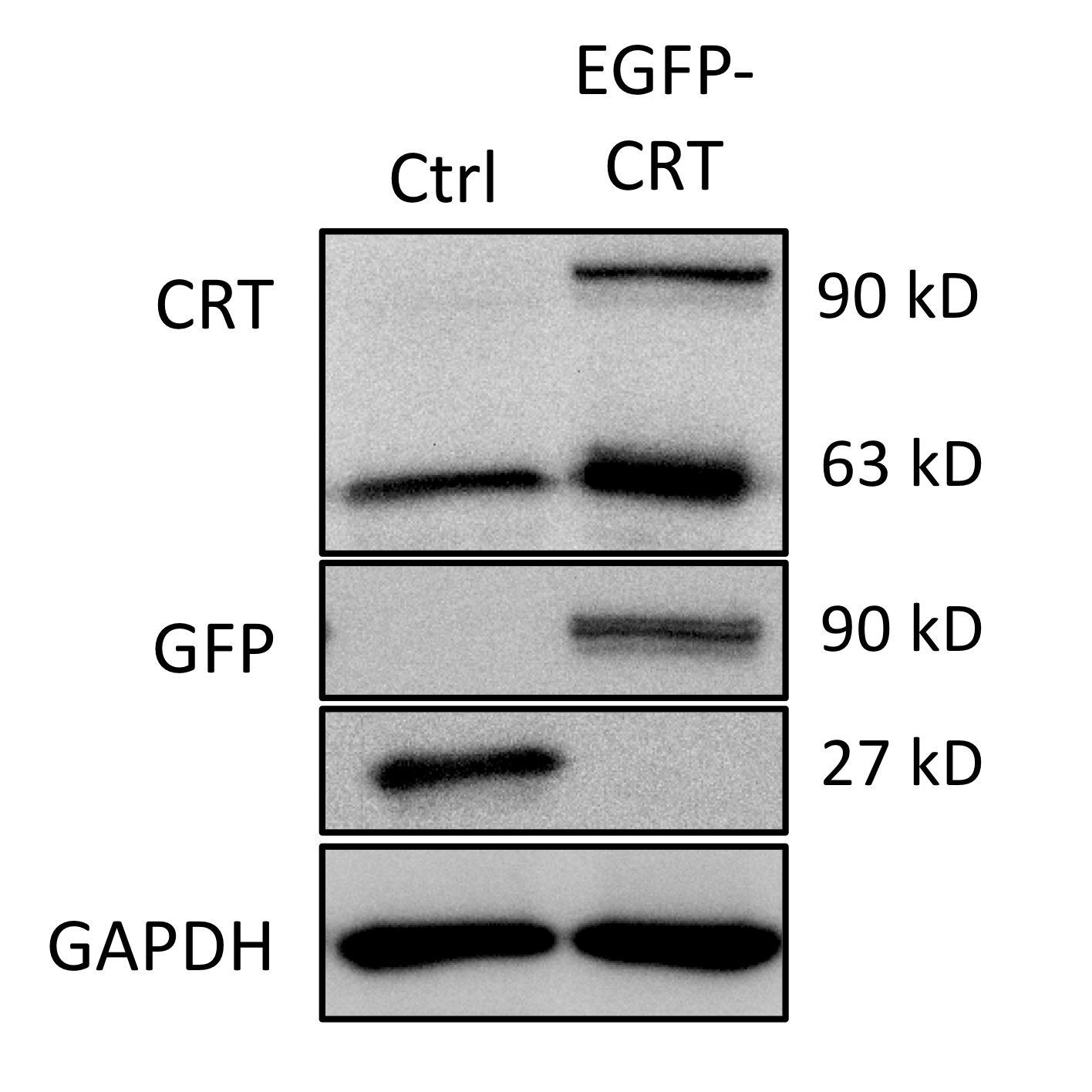

Supplement: S2 Fig — AGS cells were transfected with pEGFP-CRT or pEGFP-C1 control to generate CRT overexpression AGS cell. Western blot analysis demonstrated the endogenous CRT (63 kD), overexpressed EGFP (27kD), and EGFP-CRT (90kD). Data are presented as mean± SD for three independent experiments. (TIFF) [file pone.0225107.s002.tiff]

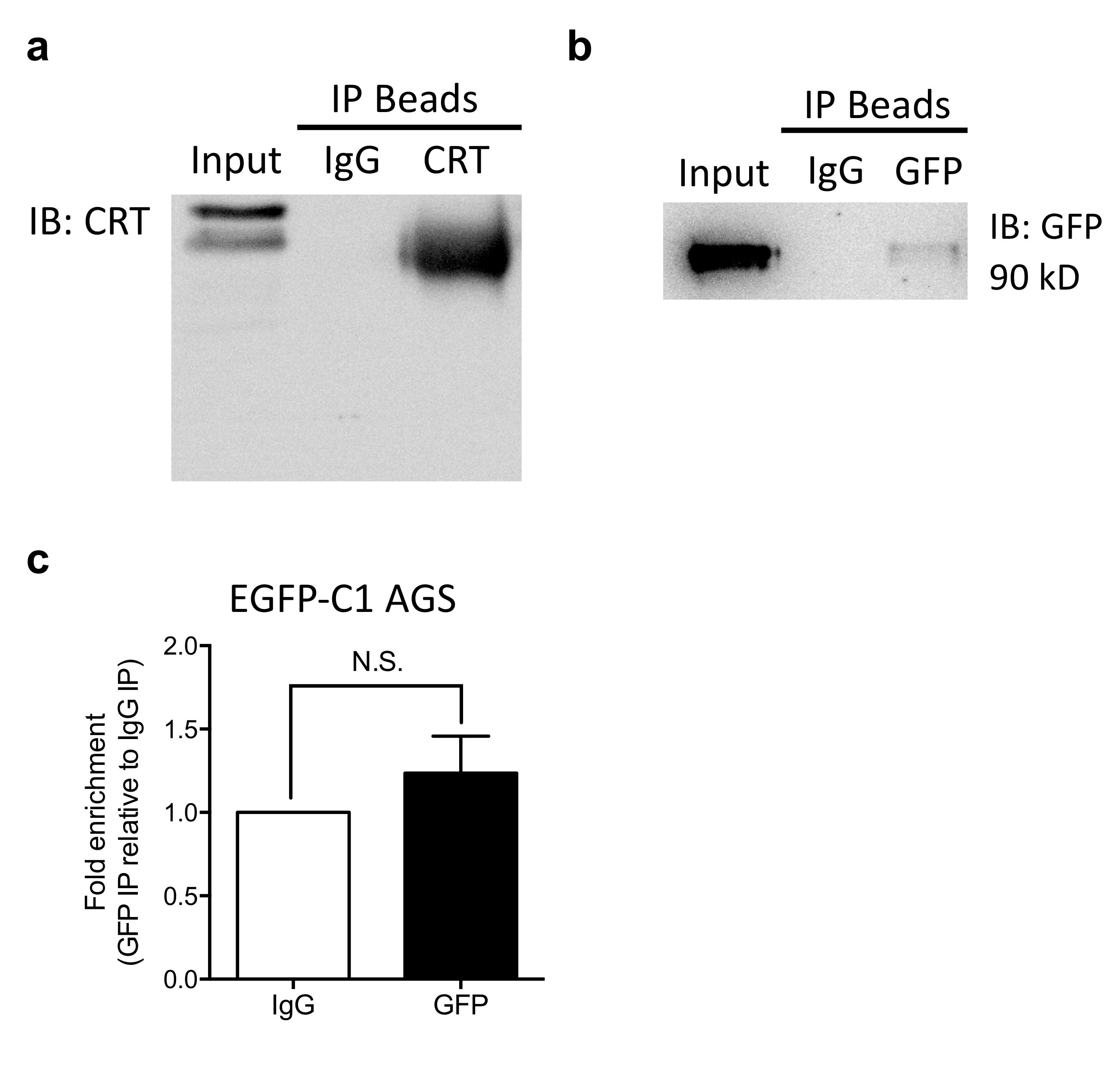

Supplement: S3 Fig — (a) AGS cell lysate was immunoprecipitated (IP) with either CRT or IgG control antibodies, followed by immunoblotting (IB) with anti-CRT antibodies to evaluate the pull-down specificity. (b) EGFP-CRT AGS cell lysate was IP with either GFP or IgG control antibodies. Western blot analysis demonstrated the pull-down specificity of overexpressed EGFP-CRT (90 kD). (c) EGFP-C1 control AGS cell lysate was incubated with either GFP or IgG control antibodies, followed by RNAIP. The enrichment of VEGF-A mRNA was normalized to the total amount VEGF-A of input and then compared to the levels in the IgG control. Real-time qPCR showed no significant change from GFP IP than control IgG IP. (TIFF) [file pone.0225107.s003.tiff]

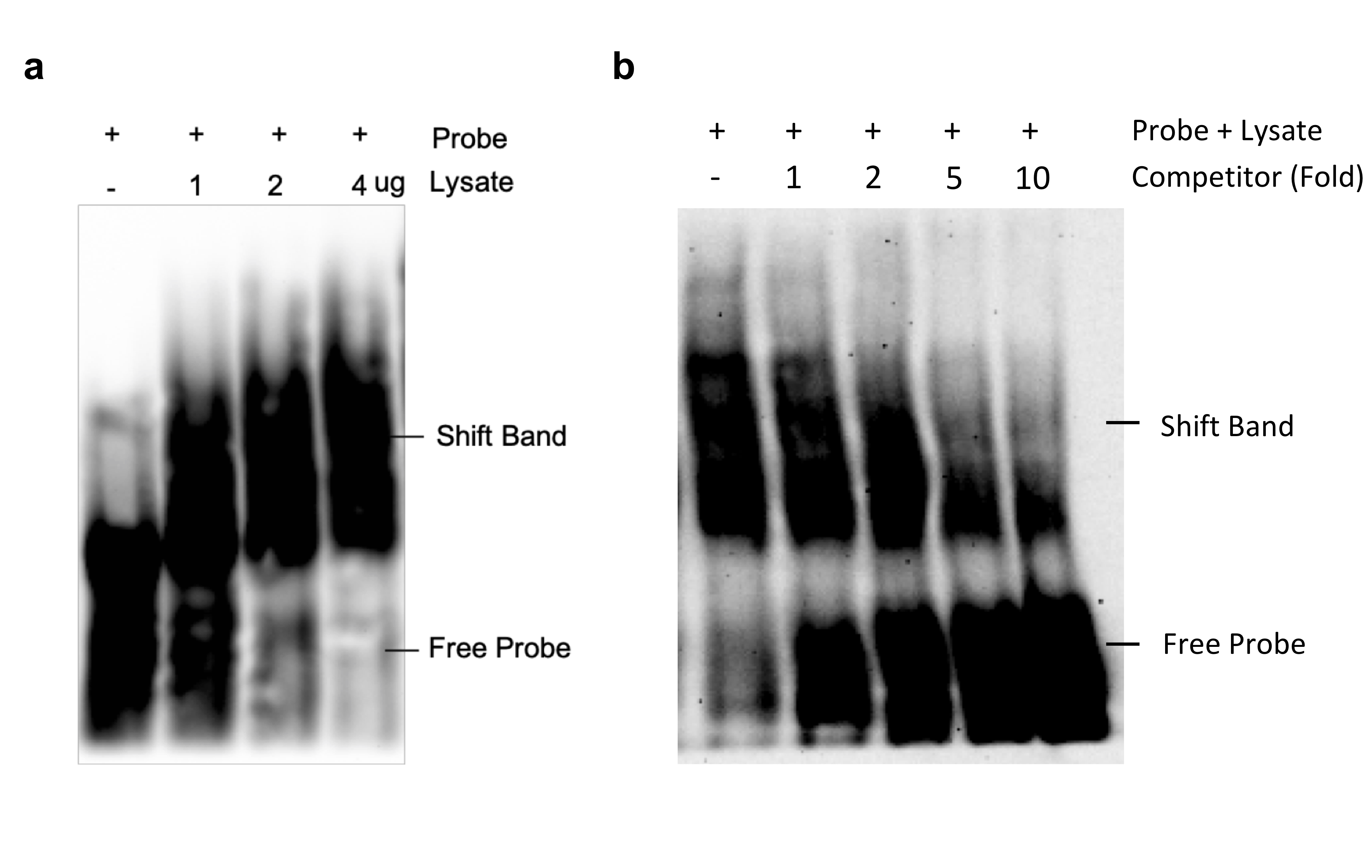

Supplement: S4 Fig — (a) Biotin labeled RNA probe was incubated with different concentration of AGS protein lysate (form 1 to 4 μg). The shift band was indicated a specific RNA-protein complex. (b) 1.25 pmol biotin labeled ARE containing VEGF-A RNA probe was incubated with AGS protein lysate in the absence or presence of 1-10-fold unlabeled probe (same sequence competitor). (TIFF) [file pone.0225107.s004.tiff]

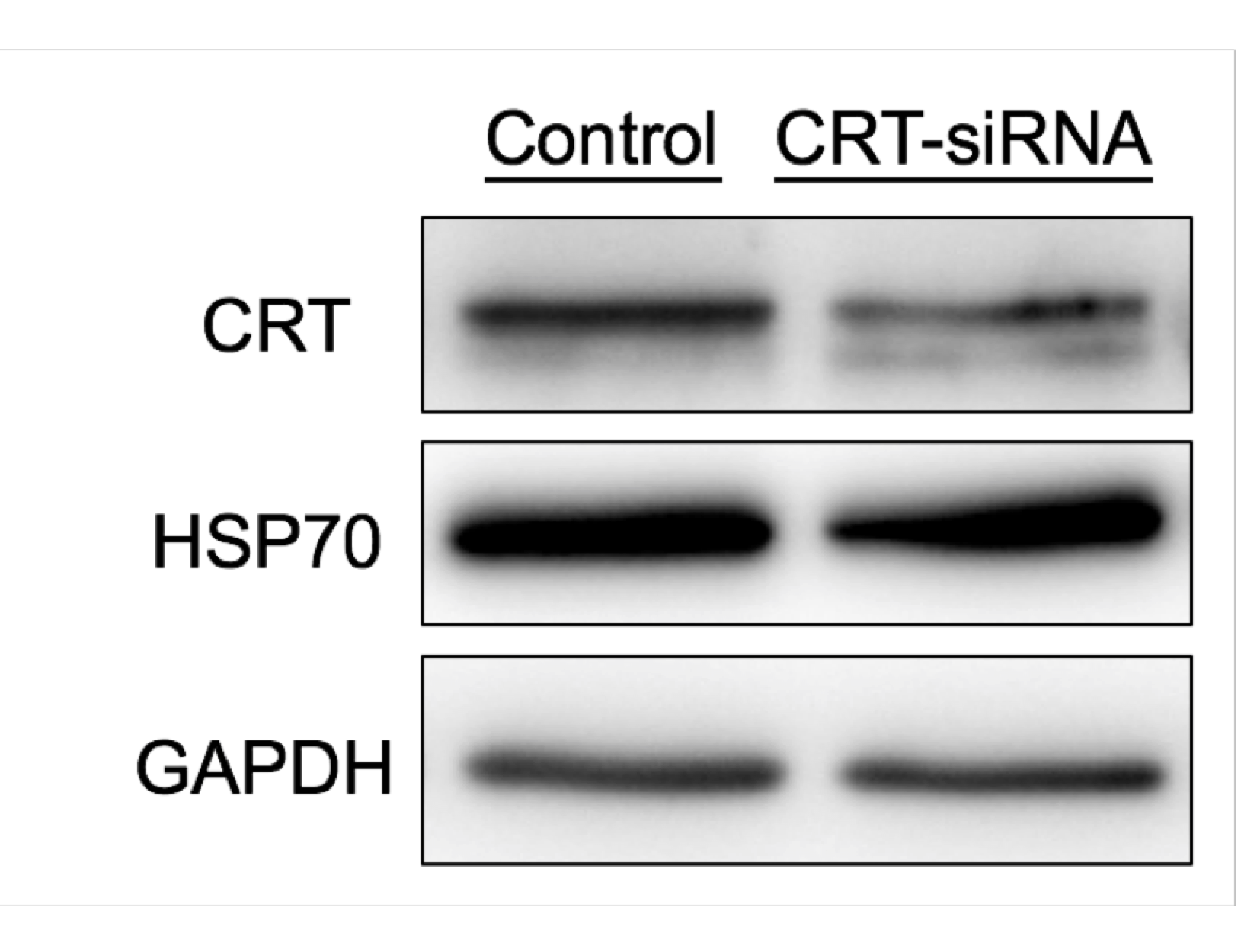

Supplement: S5 Fig — MKN45 cells were transfected with control or CRT-siRNA to generate CRT knockdown cells. Western blot analysis demonstrated the protein level of CRT and HSP70 in the MKN45 cells. Human GAPDH was used as a loading control. (TIFF) [file pone.0225107.s005.tiff]
