# Supplementary material for: Calreticulin regulates vascular endothelial growth factor-A mRNA stability in gastric cancer cells
Source: PLoS One. 2019 Nov 14;14(11):e0225107. doi: 10.1371/journal.pone.0225107 (PMC6855450; doi:10.1371/journal.pone.0225107)

S6 Fig: Full-length membranes and gels

Full-length membranes for Fig. 1a

Stage I, II -1, anti-CRT

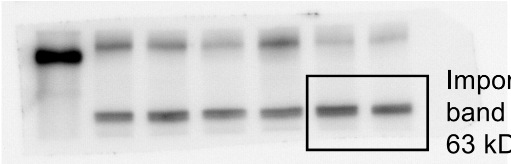

Important band 63 kD

Stage I, II -1, anti-GAPDH

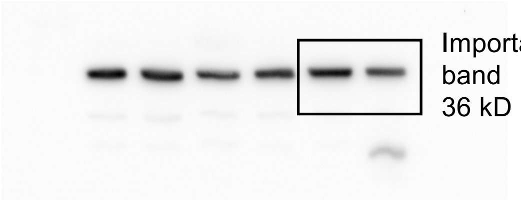

Important band 36 kD

Stage I, II -1, anti-VEGFA

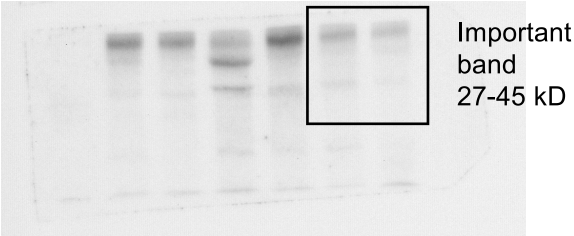

Important band 27-45 kD

Stage I, II -2, anti-CRT

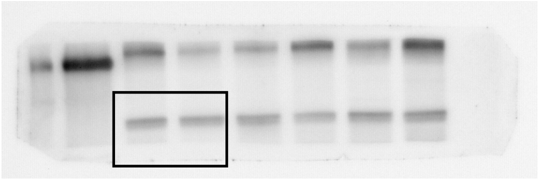

Stage I, II -1, anti-GAPDH

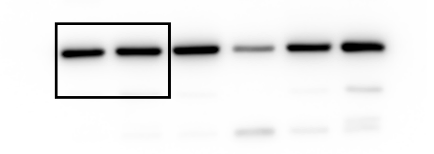

Stage I, II -2, anti-VEGFA

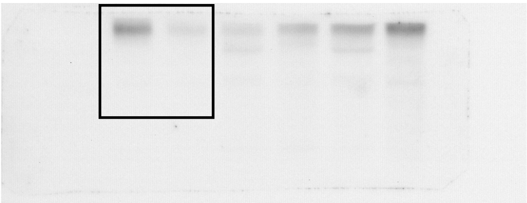

Stage III, IV -1, anti-CRT

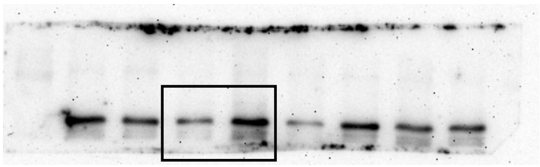

Stage III, IV -1, anti-GAPDH

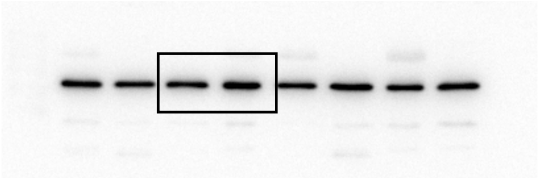

Stage III, IV -1, anti-VEGFA

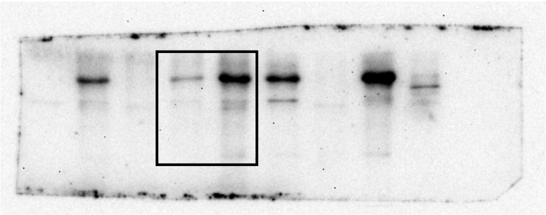

Stage III, IV -2, anti-CRT

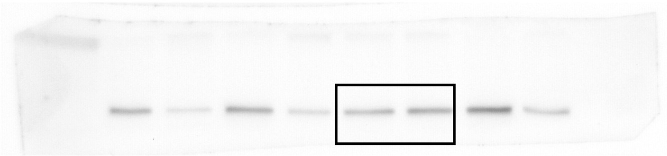

Stage III, IV -2, anti-GAPDH

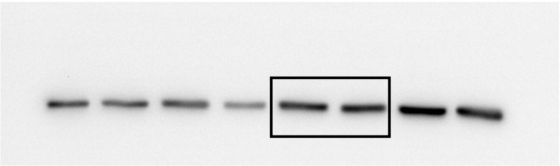

Stage III, IV -2, anti-VEGFA

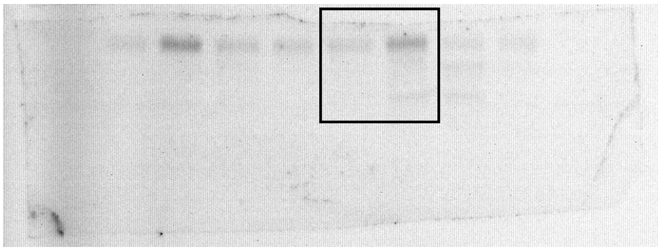

Full-length membranes for Fig. 3a

anti-CRT

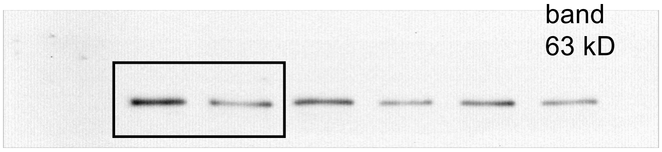

Important band 63 kD

anti-VEGFA

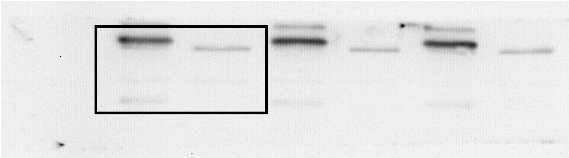

Important band 27-45 kD

anti-GAPDH

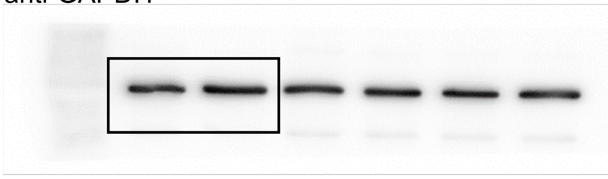

Important band 36 kD

Full-length membranes for S1a Fig.

anti-CRT

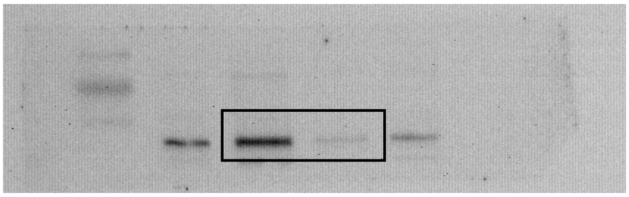

anti-GAPDH

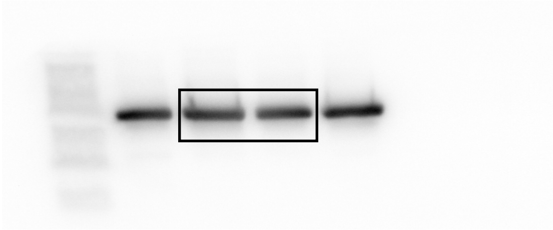

anti-VEGFA

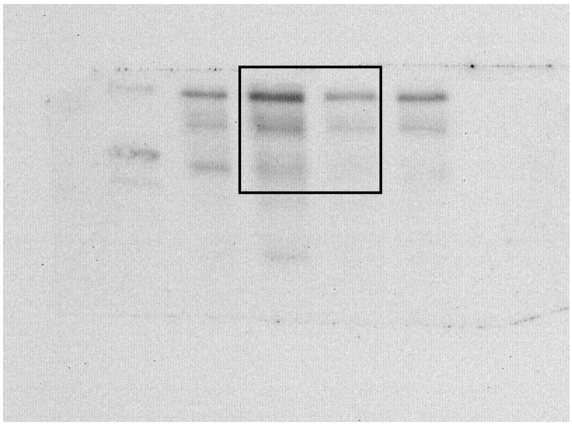

Full-length membranes for S2a Fig.

anti-CRT

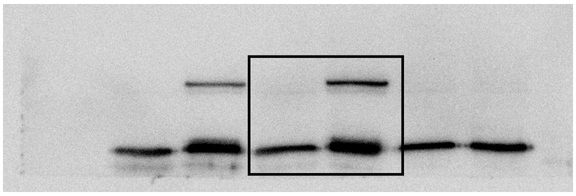

anti-VEGFA

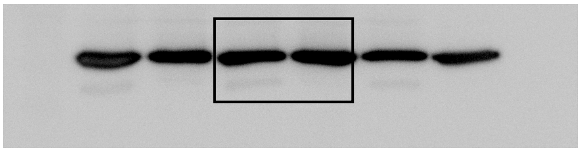

anti-GFP

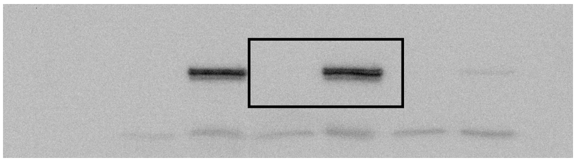

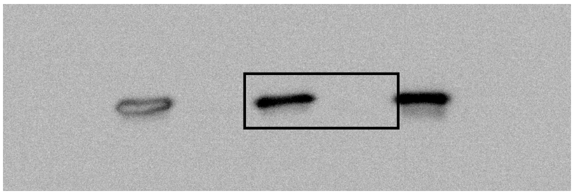

Full-length membranes for Fig 4

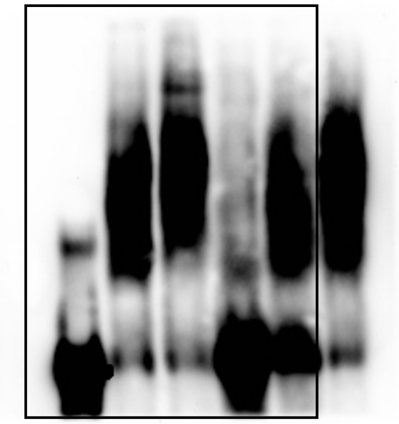

Full-length membranes for S3 Fig.

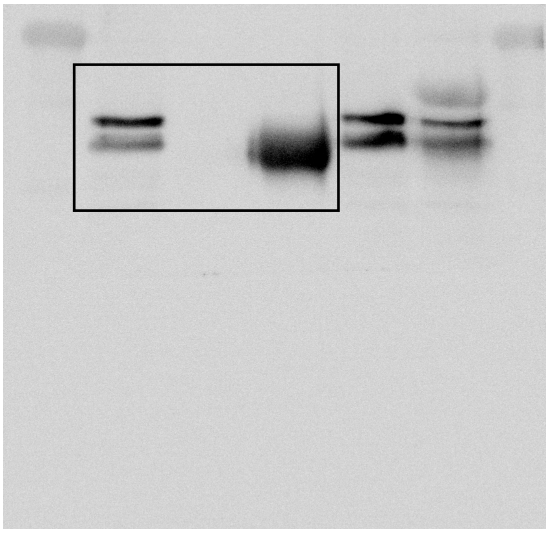

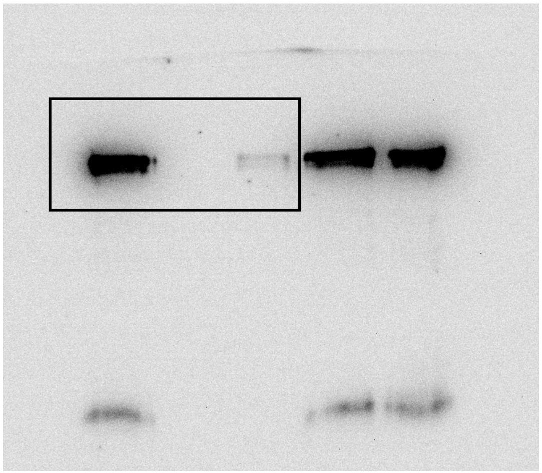

Full-length membranes for S4 Fig.

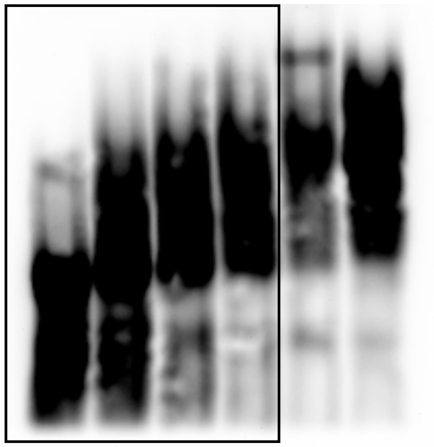

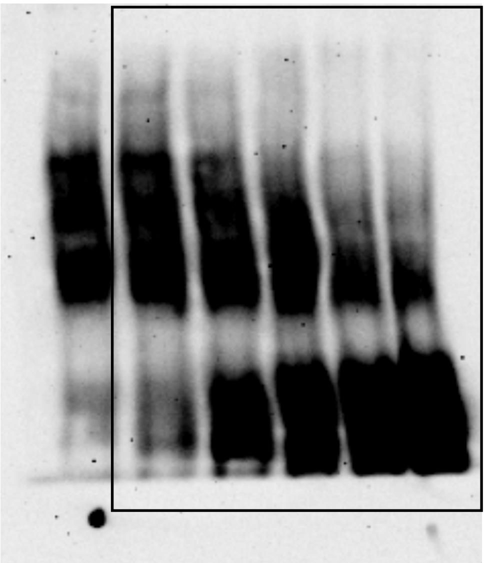

Full-length membranes for S5 Fig.

anti-CRT

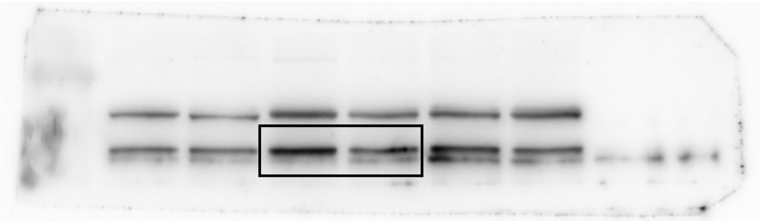

anti-HSP70

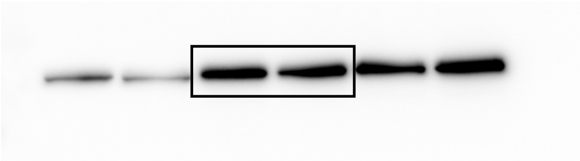

anti-GAPDH

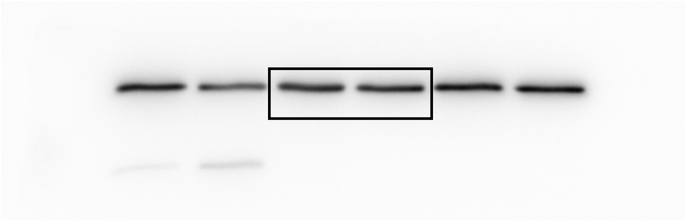

Supplement: S6 Fig — (PDF) [file pone.0225107.s006.pdf]
